# Exosomal Circular RNA RNA-seq Profiling and the Carcinogenic Role of Exosomal circ-CYP24A1 in Cutaneous Squamous Cell Carcinoma

**DOI:** 10.3389/fmed.2021.675842

**Published:** 2021-05-24

**Authors:** Zheng Zhang, Hao Guo, Wenjia Yang, Jiuhong Li

**Affiliations:** Department of Dermatology, Key Laboratory of Immunodermatology, The First Hospital of China Medical University, Shenyang, China

**Keywords:** exosomes, circular RNA, cutaneous squamous cell carcinoma, circ-CYP24A1, progression

## Abstract

**Objective:** Aberrantly expressed exosomal circular RNAs (circRNAs) have been reported in various human cancers. Nevertheless, it remains elusive in cutaneous squamous cell carcinoma (cSCC). Herein, based on RNA-seq, we systematically uncovered the expression and implication of exosomal circRNAs in cSCC.

**Methods:** Plasma exosomes derived from cSCC and healthy subjects were characterized by nanoparticle tracking analysis (NTA), transmission electron microscopy (TEM), and western blot. Differentially expressed exosomal circular RNAs (circRNAs) were screened by RNA-seq analysis, which were validated by RT-qPCR. Among them, the biological structure of circ-CYP24A1 was validated by Sanger sequencing and RNase R digestion. Si-circ-CYP24A1 was transfected into exosomes, followed by incubation with A431 and SCL-1 cells. Then, viability, apoptosis, migration, and invasion were evaluated by CCK-8, TUNEL staining and migration assays.

**Results:** This study identified 25 up- and 76 down-regulated exosomal circRNAs in cSCC than healthy subjects. Among them, circulating circ-CYP24A1 was confirmed to be up-regulated in cSCC. Circ-CYP24A1 had a covalently closed circular structure and was not sensitive to RNase R digestion. After incubation with si-circ-CYP24A1-transfected exosomes, proliferation, migration, and invasion were lowered while apoptosis was enhanced in A431 and SCL-1 cells. Meanwhile, si-circ-CYP24A1-transfected exosomes significantly decreased the expression of downstream targets CDS2, MAVS, and SOGA in cSCC cells.

**Conclusion:** Collectively, our study identified that targeting exosomal circ-CYP24A1 could suppress cSCC progression by weakening tumor malignant behaviors, which might provide a promising therapeutic target and non-invasive diagnostic biomarker for cSCC.

## Introduction

Cutaneous squamous cell carcinoma (cSCC), a common malignant tumor, is representative of 20-50% all skin tumors, which shows a rapidly increased tendency in incidence globally (1). This malignancy has high risks of local recurrence, metastases as well as death (2). Although the outcomes of cSCC patients are acceptable, advanced cSCC is in relation to terrible morbidity as well as mortality (3). Most of cSCC subjects can be cured through complete surgical excision, but some will experience metastasis (4). The pathogenesis of cSCC has not yet been elucidated, so exploring the molecular mechanisms of its occurrence and progression may help prolong the survival time of patients.

Circular RNA (circRNA) is a non-coding RNA with a covalently closed circular structure without 5′ caps and 3′ poly tails (5). In recent years, it has gradually become a hot spot in molecular biology and clinical diagnostics. The expression of circRNAs in different species, tissues, and cells is different, with tissue specificity (6). Furthermore, circRNAs are not sensitive to nucleases and are more stable than linear RNAs, which make circRNAs have obvious advantages in the development and application of new clinical diagnostic markers (7). CircRNAs can also be used as molecular sponge of miRNAs to inhibit the activity of miRNAs, and has the function of regulating gene transcription and binding RNA binding proteins (8). The extensiveness, conservation, and tissue specificity all indicate that circRNAs are expected to become new tumor markers and potential targets, providing new directions for tumor diagnosis and targeted therapy (9). At present, there are few research reports on circRNAs in cSCC, and there is a lack of research on screening abnormally expressed circRNAs from an omics perspective.

Exosomes, extracellular vesicles secreted from eukaryotic cells (30–150 nm), are involved in cell-to-cell communication (10). Exosomes are key biological signal transmitters and carriers in pathological and physiological processes (11). Studies have shown that exosomes are released from cells through exocytosis, and then taken up by target cells, and can transfer biological signals between local or remote cells (12). Exosomes play wide roles in intercellular communication mainly through paracrine and autocrine processes, and actively act as biologically active molecules between different tissues and cells. Exosomes carry biologically active components derived from parent cells and are used as carriers for cell-to-cell communication (13). Their specific communication functions are closely related to the nature of the molecules they transport, and target cells can be modified while transferring these molecules. Recent research has verified the enrichment and stability of circRNAs in exosomes (14). Nevertheless, the clinical implications of exosomal circRNAs are still uncharted in cSCC. Here, this study systematically analyzed the expression patterns of exosomal circRNAs in cSCC by RNA-seq and validated the carcinogenic role of exosomal circ-CYP24A1 in cSCC, which extended the precise roles and mechanisms of exosomal circRNAs.

## Materials and Methods

### Patients and Specimens

A total of five cSCC patients and five healthy subjects were recruited from The First Hospital of China Medical University (Shenyang, China) between 2018 and 2019. We collected the clinical information of each subject including maximum tumor diameter, tumor thickness, preoperative serum SCC-Ag and serum SCC-Ag 1 month after surgery. Each participant signed the informed consent and this study gained the Ethics Committee of The First Hospital of China Medical University (2018010). 5 mL of peripheral venous blood samples were drawn from each participant, which were then stored at −4°C overnight. The samples were immediately centrifuged at 2,000 g for 5 min. The upper serum samples were taken and placed in a sterile EP tube at −80°C.

### Isolation of Exosomes From Plasma and Medium

According to the specification of the exoEasy Maxi Kit (Qiagen), exosomes were isolated from plasma and cell culture medium. 1 volume of XBP buffer was added to 1 volume of samples. After mixing immediately, the sample/XBP buffer mixture was put at room temperature. The mixture was then added to the exoEasy spin column and centrifuged at 500 g for 1 min. After discarding the flow-through, the spin column was placed in the same collection tube. 10 mL XWP buffer was added to the tube and residual volume in the spin column was removed by centrifugation at 5,000 g for 5 min. All flow-through in the collection tube was discarded. The spin column was moved to a new collection tube. 400 μl XE buffer was added to the membrane and incubated for 1 min. Following centrifugation at 500 g for 5 min, the eluate was collected and re-added to the exoEasy spin column. Then, the eluate was incubated for 1 min and centrifuged at 5,000 g for 5 min. The eluate was harvested and transferred to a new collection tube.

### Nanoparticle Tracking Analysis

The pellet of exosomes after centrifugation was dissolved in 1 mL PBS. The samples were vortexed to distribute as evenly as possible and diluted to 1 × 10^12^/L exosomal suspension. 1 mL exosomal suspension was taken and placed it in a clean cuvette. A Malvern Panalytical particle size analyzer was used to detect the particle size distribution of exosomes.

### Transmission Electron Microscopy

10 μg plasma exosomes were dropped on a dedicated copper net and fixed with 1% glutaraldehyde fixative for 10 min. Then, samples were rinsed with PBS buffer. After the negative staining with 2% phosphotungstic acid, the TEM (JEM-2100) was used to observe and take pictures under a voltage of 100 kV.

### Western Blot

After resuspending plasma exosomes in a lysis buffer containing protease inhibitors, total protein was extracted for analysis. The extracted protein was added to polyacrylamide gel electrophoresis for separation and transferred to PVDF membranes. After blocking with 5% skim milk for 1 h, membranes were incubated with CD9 (1:2000; ab92726; Abcam), CD63 (1:2000; ab134045; Abcam), TSG101 (1:1000; ab125011; Abcam) overnight in a refrigerator at 4°C. After washing with PBS buffer, the secondary antibody was incubated for 1 h. The chemiluminescence method was utilized to develop color.

### CircRNA RNA-Seq Analysis

In line with the QIAzol kit procedure, total exosomal RNAs were extracted from three pairs of cSCC patients and healthy subjects. After the purity and concentration test met the standards, Shanghai Yunxu Biotechnology Co., Ltd. applied RNA-seq technology to detect the circRNA expression profile of exosomes. Briefly, ribosomal RNAs were removed with NEBNext rRNA Depletion Kit (New England Biolabs, Inc., Massachusetts, USA) and circRNAs were enriched in extracted exosomal RNAs. After constructing the RNA library with NEBNext^®^ Ultra™ II Directional RNA Library Prep Kit (New England Biolabs, Inc., Massachusetts, USA), libraries were controlled for quality and quantified through the BioAnalyzer 2100 system (Agilent Technologies, Inc., USA). Library sequencing was carried out on an illumina Hiseq instrument with 150 bp paired end reads. After sequencing by Illumina HiSeq 4000 sequencer, paired-end reads were harvested. Q30 was used for quality control. Cutadapt software (v1.9.3) was employed to trim the 3'end and delete the poor-quality reads (15). By STAR software (v2.5.1b), the obtained high-quality reads were compared with the reference genome (UCSC HG19) (16). The obtained circRNAs were identified through the DCC software (v0.4.4) that had high predictive accuracy and sensitivity (17). The edgeR software (v3.16.5) was applied for standardization for raw junction reads with TMM method, followed by log2 conversion (18). CircRNAs were annotated by the Circ2Trait (19) and CircBase databases. Differentially expressed circRNAs were screened between cSCC and healthy subjects with |fold change (FC)| ≥2 and *p*-value ≤ 0.05. Gene Ontology (GO) and Kyoto Encyclopedia of Genes and Genomes (KEGG) enrichment analyses were presented for differentially expressed circRNAs-associated genes *via* clusterProfiler package (20). Terms with *p*-value ≤ 0.05 were significantly enriched. RNA-seq data have been uploaded to the GEO database (accession number: GSE136113).

### RT-qPCR

Total RNA was extracted from plasma and cells, which was reverse transcribed into cDNA via PrimerscriptTMRT reverse transcription kit. RT-qPCR was presented with SYBR Premix Ex Taq^TM^ (Takara, Beijing, China). Primers were synthesized by Xiamen Antihela Biotechnology Co., Ltd. (Fujian, Xiamen, China), as listed in Table 1. The relative expression levels were determined with 2^−ΔΔ*CT*^ method.

**Table 1:** The primer sequences of RT-qPCR.

**Targets**	**Sequences (5^′^-3^′^)**
Circ-CYP24A1	TTTGCCAGCGATAATACG (forward)
	CCTGGTTTCATTAGTTTCTTT (reverse)
Circ-DNA2	GGCACCAGCATAGCCAGT (forward)
	CCAGGCGCTTTTCACAGT (reverse)
circ-SYNE2	TGCAAGAACTTTAATGACTGGT (forward)
	TTGCAGGTGGTGTTCAAGAA (reverse)
CYP24A1	AGTCTAATGTGGATTCTCT (forward)
	CGTAAGCCTCATAGATTC (reverse)
Circ-ALDH3A2	CTGTTGCTCACTTTCCTGGG (forward)
	TGACTTCCTGACTGTACACATTG (reverse)
Circ-LRBA	CCTTGCCCACCAACTTCA (forward)
	AGCCATTTTCCATGCAGC (reverse)
Circ-SENP2	ACAGCTGAATGGGAGTGATTG (forward)
	GTGGCAGCACAGAACCTTC (reverse)
Circ-HLA-B	ACTACAACCAGAGCGAGGAC (forward)
	GTAATCCTTGCCGTCGTAGG (reverse)
CDS2	TCTCTATCTAATAGGATTCTG (forward)
	TTACAACAATCAGCAATG (reverse)
MAVS	CTATAAGTATATCTGCCGCAATT (forward)
	AGTCGATCCTGGTCTCTT (reverse)
SOGA1	AAAGCATAAATCGGGCAACTC (forward)
	CTCCTCAATCTCGTCCTTCTC (reverse)
GAPDH	GGCCTCCAAGGAGTAAGACC (forward)
	AGGGGAGATTCAGTGTGGTG (reverse)

### Sanger Sequencing

2% agarose gel was prepared for this study. 2 g agar powder was poured into a glass bottle, and 100 mL 1X TAE solution was added to the bottle. Then, the bottle was repeatedly heated in a microwave oven and boiled until the agar powder was completely dissolved. The glass bottle was cooled to 60°C. Afterwards, 1 μl Red was added according to the instructions. The agarose gel liquid was poured into the tank and let it stand at room temperature until the gel was completely solidified for 40 min. The comb was then gently and slowly pull out. The gel was put into the electrophoresis tank in the correct way and 1XTAE buffer was added to it. PCR product and DNA maker were added to the sample tank. The voltage was set to 100 V, and the electrophoresis was about 30 min. After the electrophoresis was over, the gel was taken out. The gel imaging system was observed and the corresponding PCR band was cut out for Sanger sequencing.

### Cell Culture and Transfection

A431 cells and SCL-1 cells were purchased from the American Type Culture Collection (ATCC). The cells were cultured in DMEM medium containing 10% fetal bovine serum, 100 U/mL streptomycin and penicillin at 37°C and 5% CO_2_. The siRNAs were designed and synthesized by Hanheng Biological Technology Co., Ltd. (Shanghai, China). The siRNAs against circ-CYP24A1 were as follows: circ-CYP24A1, 5′-GAUAAUACGCCUCAGGGAATT-3′ (sense), 5′-UUCCCUGAGGCGUAUUAUCTT-3′ (antisense); si-negative control (si-NC), 5′- UUCUCCGAACGUGUCACGUTT-3′ (sense), 5′- ACGUGACACGUUCGGAGAATT-3′ (antisense). Exosomes were separated from the supernatant of cultured A431 cells and SCL-1 cells. Before harvesting the culture medium, A431 cells and SCL-1 cells were washed twice with PBS, and then cultured for 48 h. The supernatant was harvested and centrifuged at 2,000 g for 30 min at 4°C, 10,000 g for 30 min, and 100,000 g for 70 min. The exosomes were washed once with PBS, then ultracentrifuged again at 100,000 g for 70 min. After characterization of exosomes, the siRNAs were transfected into exosomes via Exosome Transfection Kit. Then, transfected exosomes were incubated with A431 cells or SCL-1 cells for 48 h. RT-qPCR was used for examining the expression of circ-CYP24A1.

### RNase R Digestion

Total RNA was extracted from A431 and SCL-1 cells. They, samples were incubated with 6 units of RNase R for 15 min at 37°C. The expression of circ-CYP24A1 and CYP24A1 was detected through RT-qPCR.

### Tracer Experiment

2 μg exosomes derived from A431 cells and SCL-1 cells were taken and resuspended in 1 mL Diluent C. 4 μL PKH67 was added to 1 mL Diluent C to prepare the dye. 1 mL of exosomes were mixed with 1 mL dye suspension for 5 min. 2 mL of serum was added to stop staining. The medium was changed with the complete medium containing PKH67-labeled exosomes. Then, cells were fixed with paraformaldehyde for 40 min. After staining A431 cells and SCL-1 cells with Hoechst for 10 min, they were washed twice with PBS. Then, 20 μL anti-fluorescence mounting solution was added to the slide for mounting. A laser confocal microscope was used to observe the interaction between exosomes and A431 cells and SCL-1 cells.

### Cell Counting Kit-8assay

A431 cells and SCL-1 cells were inoculated into a 96-well plate (1,000 cells/well). After co-incubating the transfected exosomes, 100 μl CCK-8 (Dojindo, Kumamoto, Japan) was added. A microplate reader was utilized to detect the absorbance at 0, 24, 48, 72, and 96 h. Six multiple holes were set in each group.

### Terminal Deoxynucleotidyl Transferase dUTP Nick-End Labeling Assay

A431 cells and SCL-1 cells were seeded into a 96-well plate (1,000 cells/well), which were co-incubated with the transfected exosomes for 48 h. The cells were fixed with 4% paraformaldehyde for 30 min. Then, the cells were incubated with TUNEL detection solution at 37°C for 60 min in the dark. After mounting with anti-fluorescence quenching mounting solution, images were acquired under a fluorescence microscope.

### Transwell Assays

A431 cells and SCL-1 cells were cultured in a 24-well plant (5,000 cells/well). The cells were co-incubated with the transfected exosomes for 24 h. The cells were resuspended with serum-free DMEM medium. 10^4^ cells were seeded onto the upper chamber of the Transwell. For invasion assay, the bottom of the upper chamber was pre-coated with Matrigel (Corning, USA). Meanwhile, 650 μL DMEM complete medium containing 10% FBS was added to the bottom chamber. There were three replicate wells in each group. After culturing for 24 h, the cells on the inner surface of the bottom of the chamber were wiped with a cotton swab. The cells were fixed with paraformaldehyde and stained with crystal violet.

### Statistical Analyses

Statistical analyses were presented by R language and Graphpad Prism software. Correlation between circRNAs (circ-CYP24A1, circ-DNA2, and circ-ALDH3A2) and clinical features (including maximum tumor diameter, tumor thickness, pre-operative serum SCC-Ag, and serum SCC-Ag 1 month after surgery) was evaluated by Pearson test. Each experiment was repeated three times and data were expressed as the mean ± standard deviation. Comparisons between groups were presented by student's *t*-test or one-way analysis of variance. *P*-value < 0.05 indicated statistical significance.

## Results

### Characterization of Exosomes Derived From cSCC Patients' Plasma

**Figure 1:**
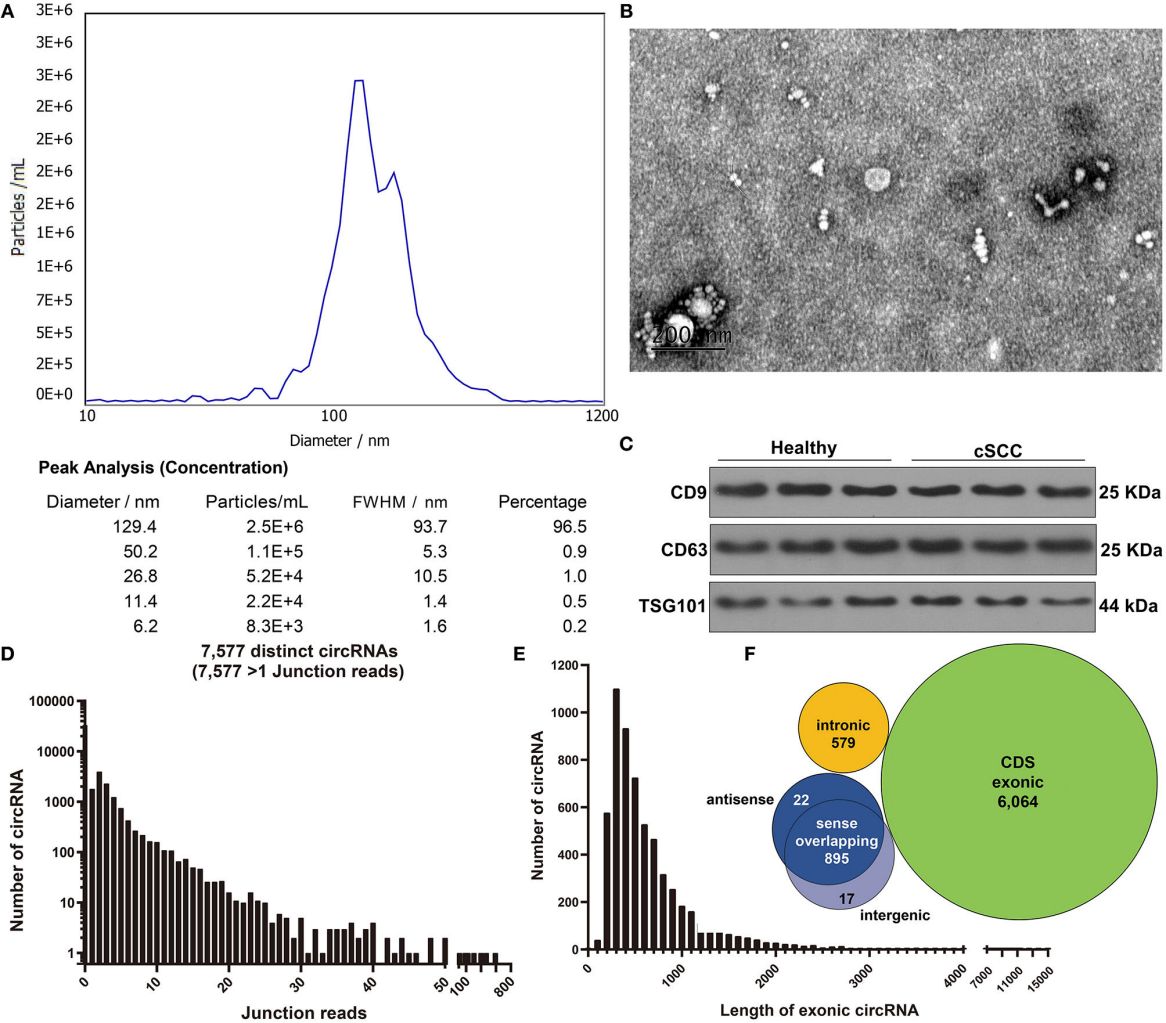
Characterization of exosomes from plasma of cSCC and healthy subjects. **(A)** NTA for detection of the particle size of isolated exosomes. **(B)** TEM for the appearance of exosomes. Bar = 200 nm. **(C)** Western blot for examining the expression of exosomal specific markers CD9 and CD63. **(D)** The junction reads of exosomal circRNAs. **(E)** The length of exonic circRNAs. **(F)** The genomic source of exosomal circRNAs.

Here, plasma samples from three paired cSCC patients and healthy individuals. Firstly, plasma exosomes were identified by employing NTA, TEM, and western blot. NTA results showed the peak size of most exosomes (96.5%) was 129.4 nm (Figure 1A). TEM analysis demonstrated that exosomes that both came from cSCC and healthy individuals had a single layer membrane structure and the appearance of exosomes was round or elliptical vesicle-like structure with a diameter of 40-100 nm, which conformed to exosomal morphology (Figure 1B). Our western blot results confirmed that exosomal specific markers CD9 and CD63 had high abundance in plasma exosomes from cSCC and healthy individuals (Figure 1C). RNA-seq technique was employed to obtain exosomal circRNAs. After image recognition and base recognition, the original reads were harvested from the Illumina HiSeq sequencer. We applied cutadapt software to obtain high-quality clean reads. By STAR software, we compared clean reads to UCSC hg19. Figure 1D showed the junction reads of identified circRNAs. A total of 7,577 exosomal circRNAs were detected by DCC software. We divided the circRNAs into both ends on exonic, intronic, intergenic and antisense circRNAs according to the alignment position of the two ends of the circRNAs. The length of both ends on exonic circRNAs was counted, as shown in Figure 1E. As expected, most exosomal circRNAs were composed of both ends on exons, followed by intergenic and antisense (Figure 1F).

### Identification of Exosomal CircRNAs and Their Functions in cSCC

**Figure 2:**
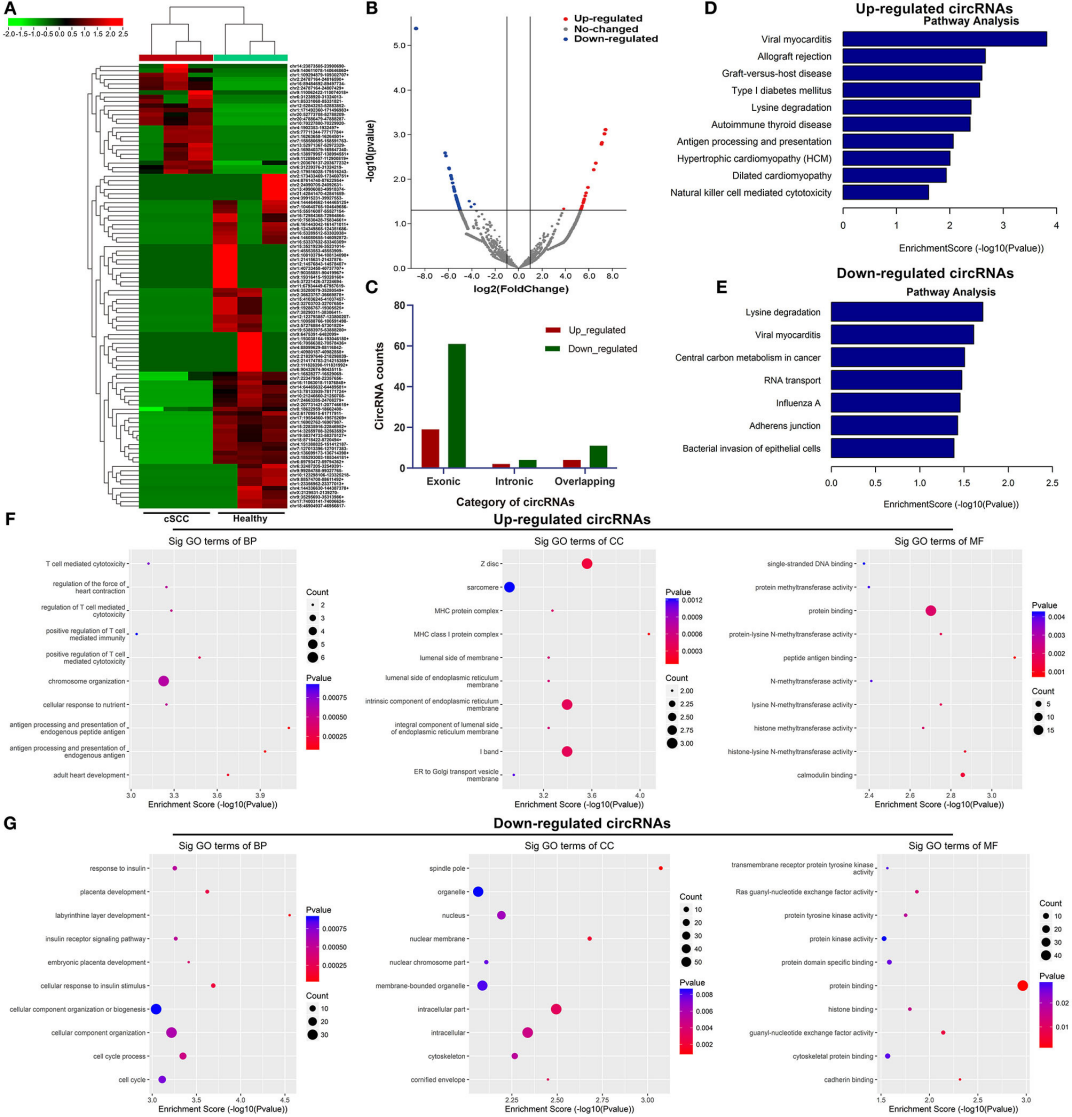
Identification of exosomal circRNAs and their functions in cSCC. **(A)** Heatmap for the up- (red) and down-regulated (green) exosomal circRNAs between cSCC and healthy individuals. **(B)** Volcano plots of up- (red) and down-regulated (blue) circRNAs between cSCC and healthy individuals. **(C)** Category of differentially expressed circRNAs. **(D,E)** KEGG pathway enrichment results of up- and down-regulated circRNAs. **(F,G)** GO annotation results of up- and down-regulated circRNAs.

Based on the standardized number of reads, logCPM value of each circRNA was calculated in each sample. We screened the differentially expressed exosomal circRNAs between cSCC and healthy samples. 25 circRNAs were up-regulated and 76 were down-regulated in cSCC compared to healthy subjects with the threshold of |FC| ≥ 2 and *p*-value ≤ 0.05 (Figures 2A,B; Supplementary Table 1). Most of them were exonic circRNAs (Figure 2C). We further annotated the host genes of these differentially expressed circRNAs to predict their functions. As a result, up-regulated circRNAs were mainly enriched in immune-related pathways like antigen processing and presentation and natural killer cell mediated cytotoxicity (Figure 2D) while down-regulated circRNAs were significantly related to central carbon metabolism in cancer, RNA transport and bacterial invasion of epithelial cells (Figure 2E). GO enrichment analysis also revealed that up-regulated circRNAs were primarily enriched in T cell mediated cytotoxicity or immunity and MHC protein complex (Figure 2F), while down-regulated circRNAs had distinct relationships with cellular component organization and cell cycle (Figure 2G).

### Construction of a circRNA-miRNA-mRNA Network for cSCC

**Figure 3:**
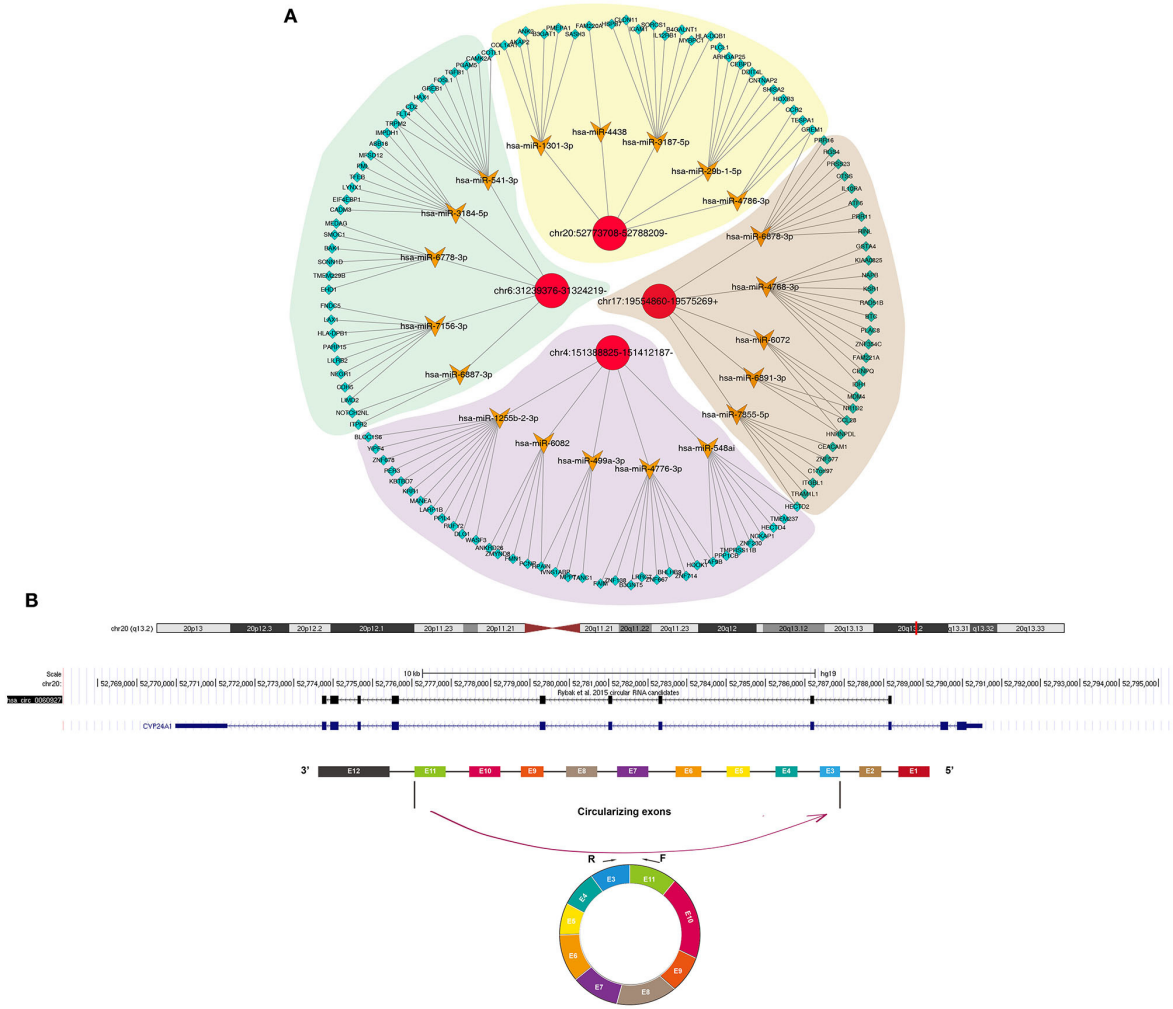
Construction of a circRNA-miRNA-mRNA network for cSCC. **(A)** The potential binding miRNAs and mRNAs for differentially expressed circRNAs. **(B)** Schematic diagram of the structure of circRNAs.

Increasing evidence highlights the roles of circRNAs as miRNA sponges. Here, we predicted the binding miRNAs of differentially expressed circRNAs through circbank database (http://www.circbank.cn/) and target mRNAs of these miRNAs by Targetscan database (http://www.targetscan.org). The potential binding miRNAs and mRNAs were detected for differentially expressed circRNAs, as shown in Figure 3A. Furthermore, we visualized the structure of circRNAs (Figure 3B).

### Clinical Characteristics of Differentially Expressed circRNAs in cSCC

**Figure 4:**
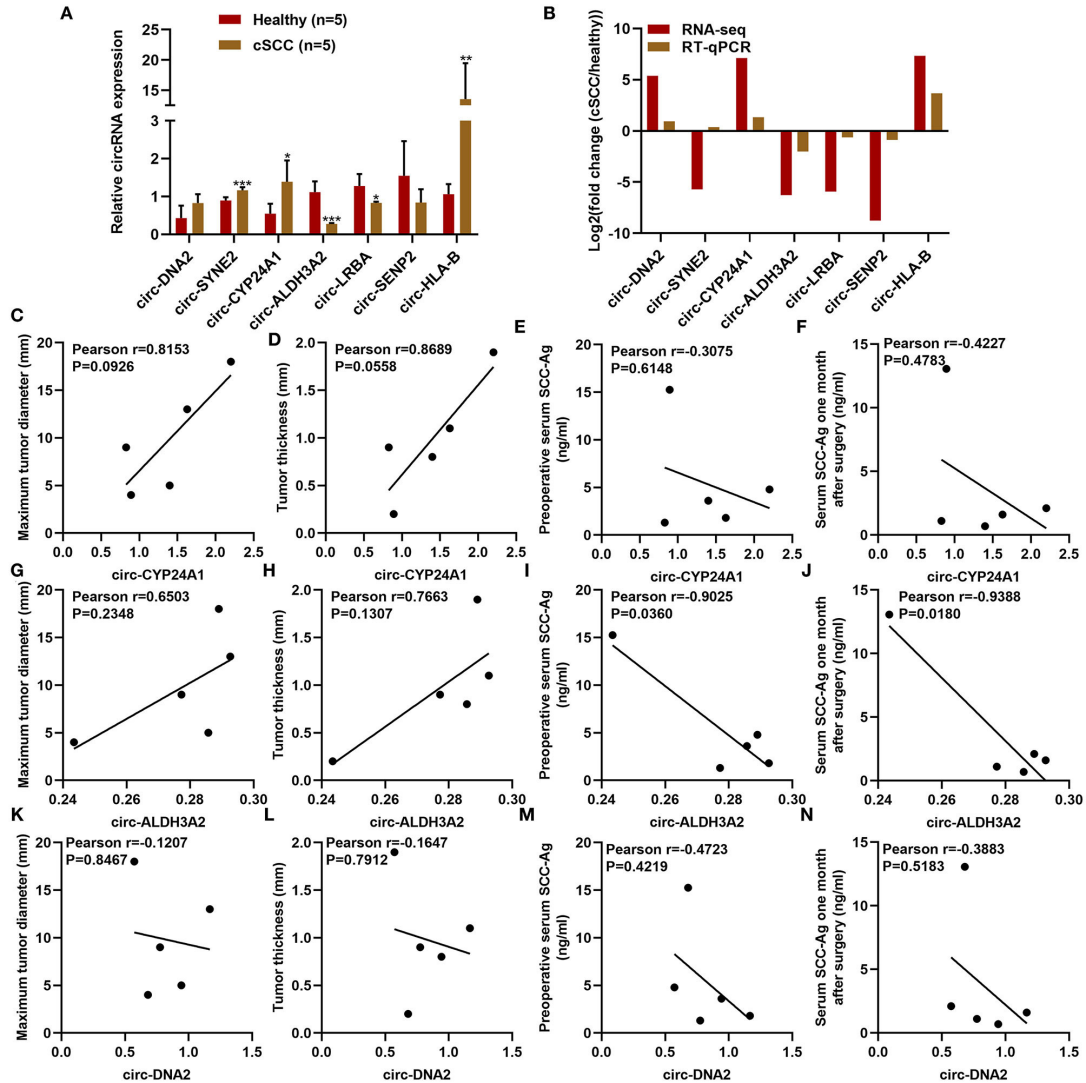
Clinical characteristics of differentially expressed circRNAs in cSCC. **(A,B)** RT-qPCR for validation of the expression of plasma circ-DNA2, circ-SYNE2, circ-CYP24A1, circ-ALDH3A2, circ-LRBA, circ-SENP2, and circ-HLA-B in 5 paired cSCC and healthy individuals. **(C-N)** The correlations of plasma **(C-F)** circ-CYP24A1, **(G-J)** circ-ALDH3A2, and **(K-N)** circ-DNA2 levels with maximum tumor diameter, tumor thickness, pre-operative serum SCC-Ag and serum SCC-Ag 1 month after surgery. *P-value < 0.05; **p-value < 0.01; ***p-value < 0.001.

RT-qPCR was used for validation of the expression of differentially expressed circRNAs including circ-DNA2, circ-SYNE2, circ-CYP24A1, circ-ALDH3A2, circ-LRBA, circ-SENP2, and circ-HLA-B in plasma samples from 5 paired cSCC and healthy individuals. Our results confirmed that plasma circ-SYNE2, circ-CYP24A1, circ-ALDH3A2, and circ-HLA-B were all significantly up-regulated in cSCC than healthy individuals while plasma circ-ALDH3A2 and circ-LRBA were both significantly down-regulated in cSCC compared to healthy subjects (Figures 4A,B). The clinical features of plasma circ-CYP24A1, circ-ALDH3A2, and circ-DNA2 were further assessed. We found that plasma circ-CYP24A1 (Figures 4C–F), circ-ALDH3A2 (Figures 4G–J) and circ-DNA2 (Figures 4K–N) levels displayed correlations to maximum tumor diameter, tumor thickness, preoperative serum SCC-Ag and serum SCC-Ag 1 month after surgery.

### Validation of the Biological Structure of Exosomal Circ-CYP24A1

**Figure 5:**
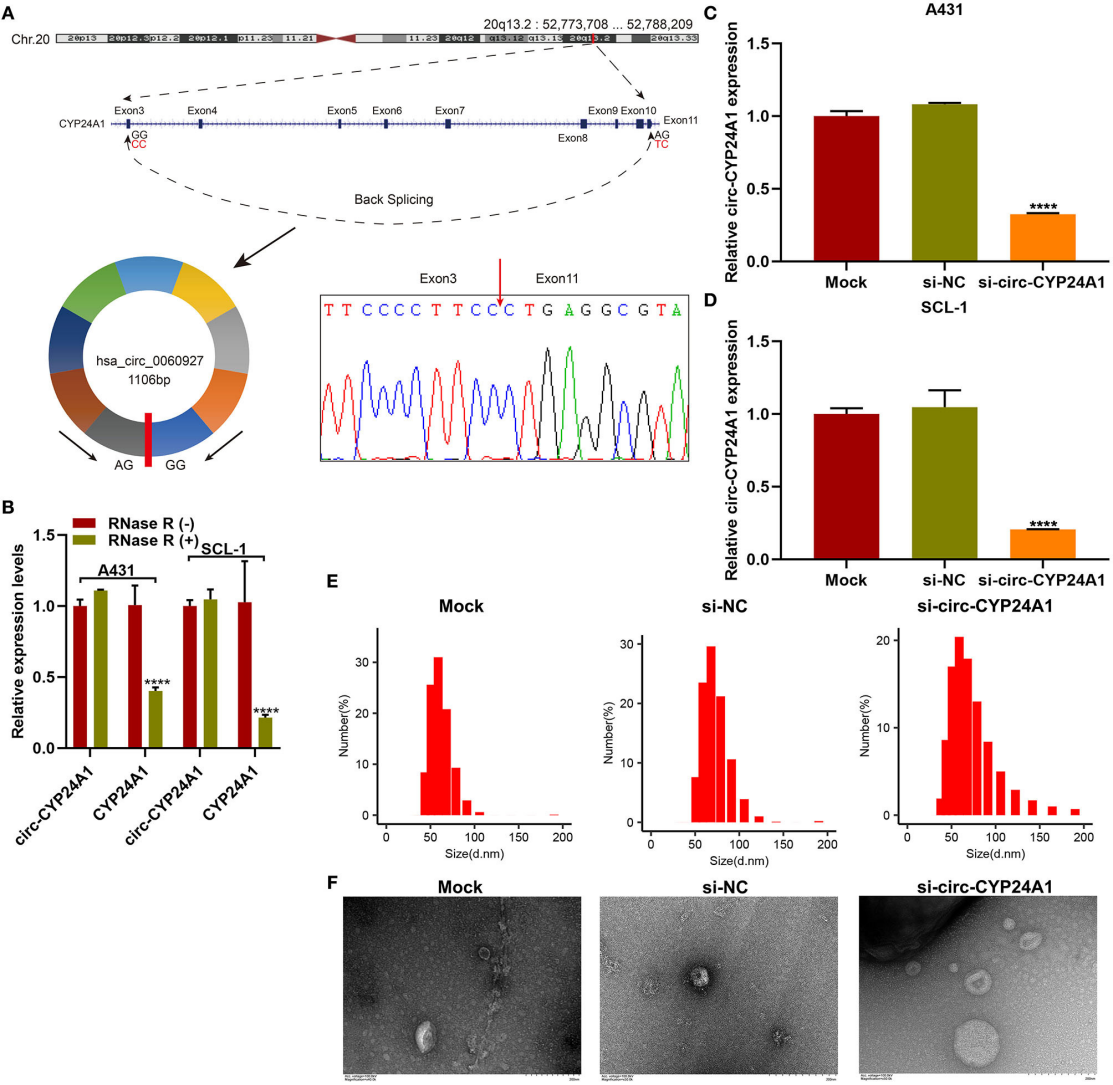
Validation of the biological structure of circ-CYP24A1. **(A)** Sanger sequencing for the circ-CYP24A1 PCR products. **(B)** RT-qPCR for the expression of circ-CYP24A1 in A431 and SCL-1 cells under RNase R (-) or RNase R (+). **(C,D)** RT-qPCR confirming the expression of circ-CYP24A1 in A431 and SCL-1 cells incubated with si-circ-CYP24A1-transfected exosomes. **(E)** NTA for detecting the particle size of transfected exosomes. **(F)** TEM for the appearance of transfected exosomes. Bar = 200 nm. ****P-value < 0.0001.

Our sanger sequencing results confirmed that circ-CYP24A1 possessed a covalently closed circular structure (Figure 5A). Furthermore, circ-CYP24A1 was not sensitive to RNase R digestion, while linear CYP24A1 was mostly digested (Figure 5B). To investigate the biological functions of exosomal circ-CYP24A1 in cSCC, A431 and SCL-1 cells were incubated with si-circ-CYP24A1-transfected exosomes. RT-qPCR results confirmed that circ-CYP24A1 was significantly lowered both in A431 cells (Figure 5C) and SCL-1 cells (Figure 5D). The exosomes transfected with si-circ-CYP24A1 were characterized by NTA (Figure 5E) and TEM (Figure 5F).

### Crosstalk Between Exosomes and cSCC Cells

**Figure 6:**
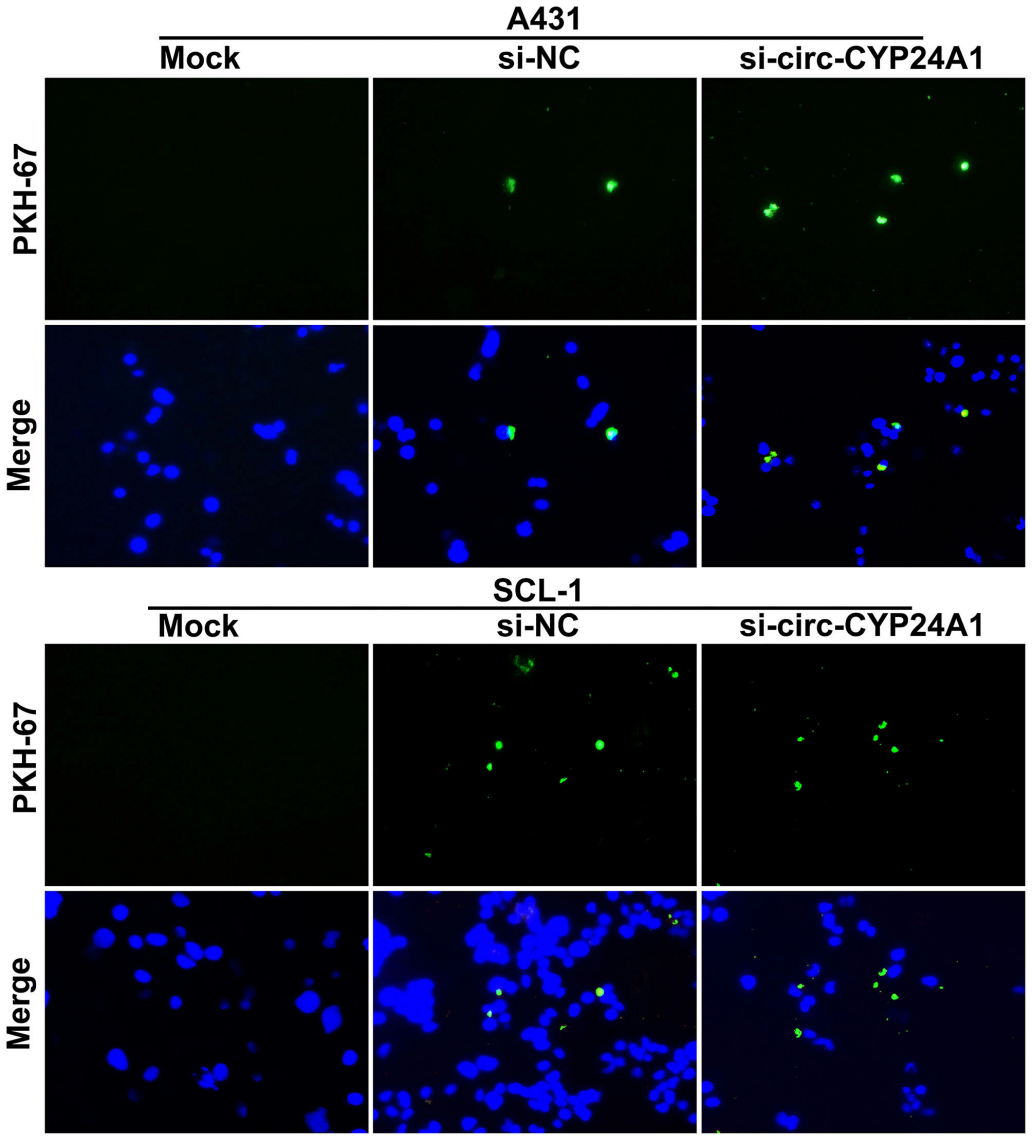
A tracer experiment for observing the uptake of exosomes carrying circ-CYP24A1 by A431 and SCL-1 cells.

To verify whether the exosomes carrying circ-CYP24A1 can be taken up by cSCC cells, this study used PKH67-labeled exosomes and co-cultured with A431 cells and SCL-1 cells. Under a laser confocal microscope, we found that exosomes carrying circ-CYP24A1 were taken up by A431 cells and SCL-1 cells (Figure 6), confirming the crosstalk between exosomes and cSCC cells.

### Exosomal Circ-CYP24A1 Knockdown Restrains Proliferation and Induces Apoptosis of cSCC Cells

**Figure 7:**
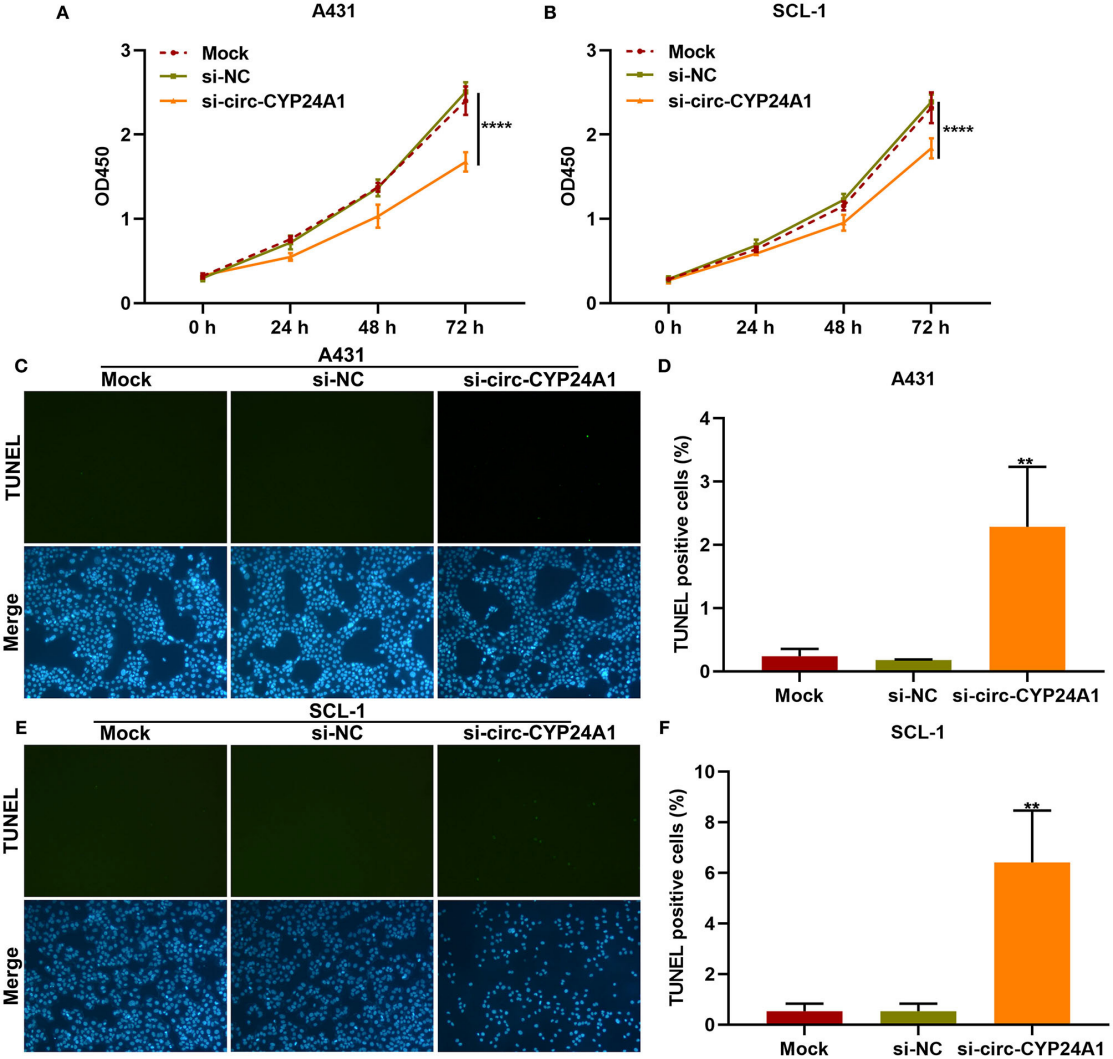
The effects of exosomal circ-CYP24A1 knockdown on proliferation and apoptosis of cSCC cells. **(A,B)** CCK-8 for the cell viability of A431 and SCL-1 cells incubated with si-circ-CYP24A1-transfected exosomes. **(C-F)** TUNEL staining for assessing the apoptosis of **(C,D)** A431 and **(E,F)** SCL-1 cells incubated with si-circ-CYP24A1-transfected exosomes. **P-value < 0.01; ****p-value < 0.0001.

We further observed whether exosomal circ-CYP24A1 affected the proliferation and apoptosis of cSCC cells. Our CCK-8 results showed that exosomes transfected with si-circ-CYP24A1 distinctly lowered the cell viability of A431 cells (Figure 7A) and SCL-1 cells (Figure 7B). As shown in TUNEL staining results, the apoptosis was markedly enhanced by exosomes transfected with si-circ-CYP24A1 in A431 cells (Figures 7C,D) and SCL-1 cells (Figures 7E,F). Thus, exosomal circ-CYP24A1 knockdown may restrain proliferation and induce apoptosis of cSCC cells.

### Exosomal Circ-CYP24A1 Knockdown Restrains Migration and Invasion of cSCC Cells

**Figure 8:**
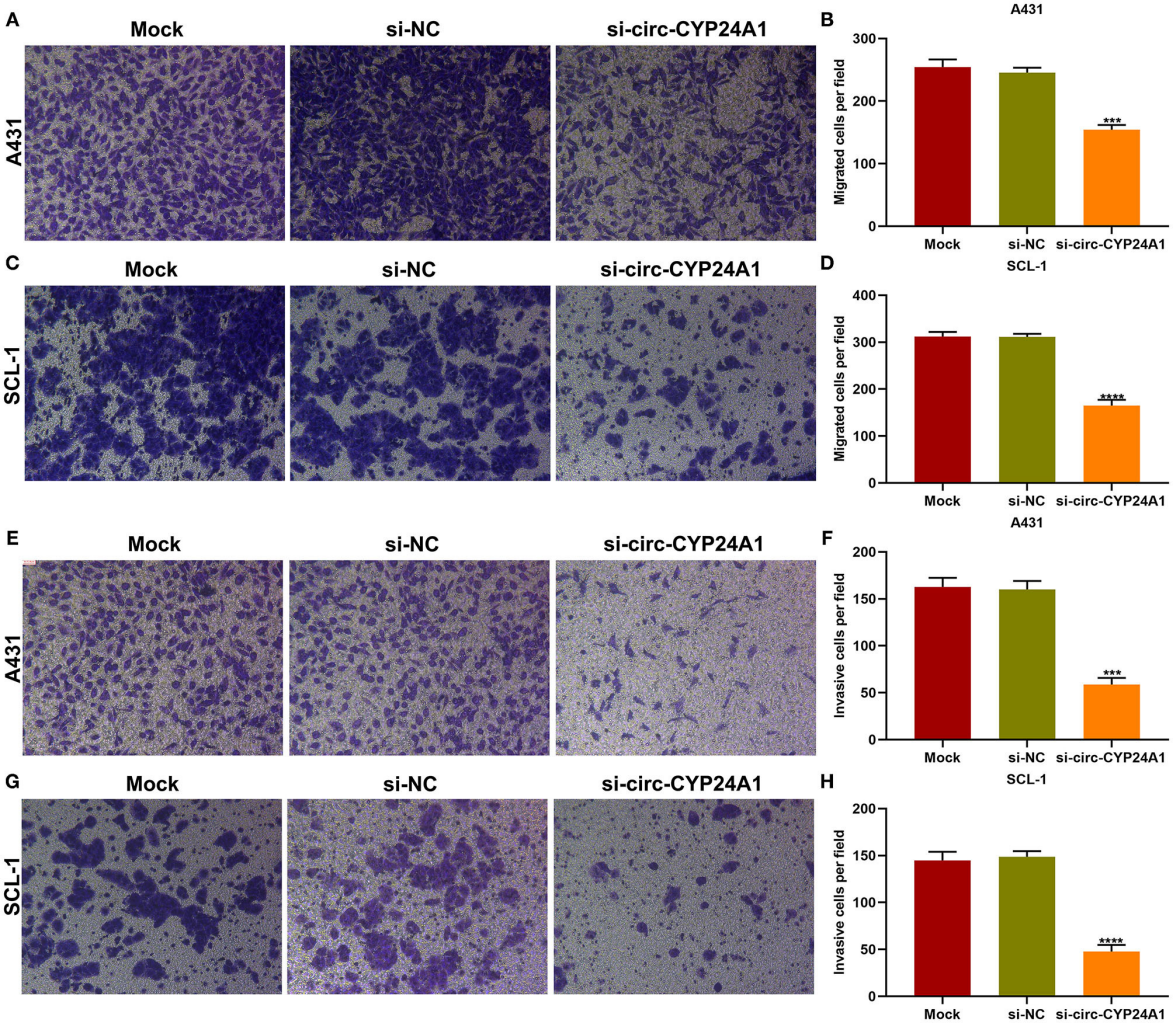
The roles of exosomal circ-CYP24A1 on migrated and invasive abilities of cSCC cells. **(A-D)** The number of migrated **(A,B)** A431 cells and **(C,D)** SCL-1 cells under incubation with si-circ-CYP24A1-transfected exosomes. **(E-H)** The number of invasive **(E,F)** A431 cells and **(G,H)** SCL-1 cells when incubated with si-circ-CYP24A1-transfected exosomes. ***P-value < 0.001; ****p-value < 0.0001.

Migration and invasion of A431 and SCL-1 cells incubated with si-circ-CYP24A1-transfected exosomes were evaluated by transwell assays. As a result, the number of migrated A431 cells (Figures 8A,B) and SCL-1 cells (Figures 8C,D) was significantly lessened after incubation with si-circ-CYP24A1-transfected exosomes. Moreover, we found that si-circ-CYP24A1-transfected exosomes markedly decreased the number of invasive A431 cells (Figures 8E,F) and SCL-1 cells (Figures 8G,H). These data demonstrated that exosomal circ-CYP24A1 knockdown may suppress migration as well as invasion of cSCC cells.

### Exosomal Circ-CYP24A1 Knockdown Lowers CDS2, MAVS and SOGA1 Expression in cSCC Cells

**Figure 9:**
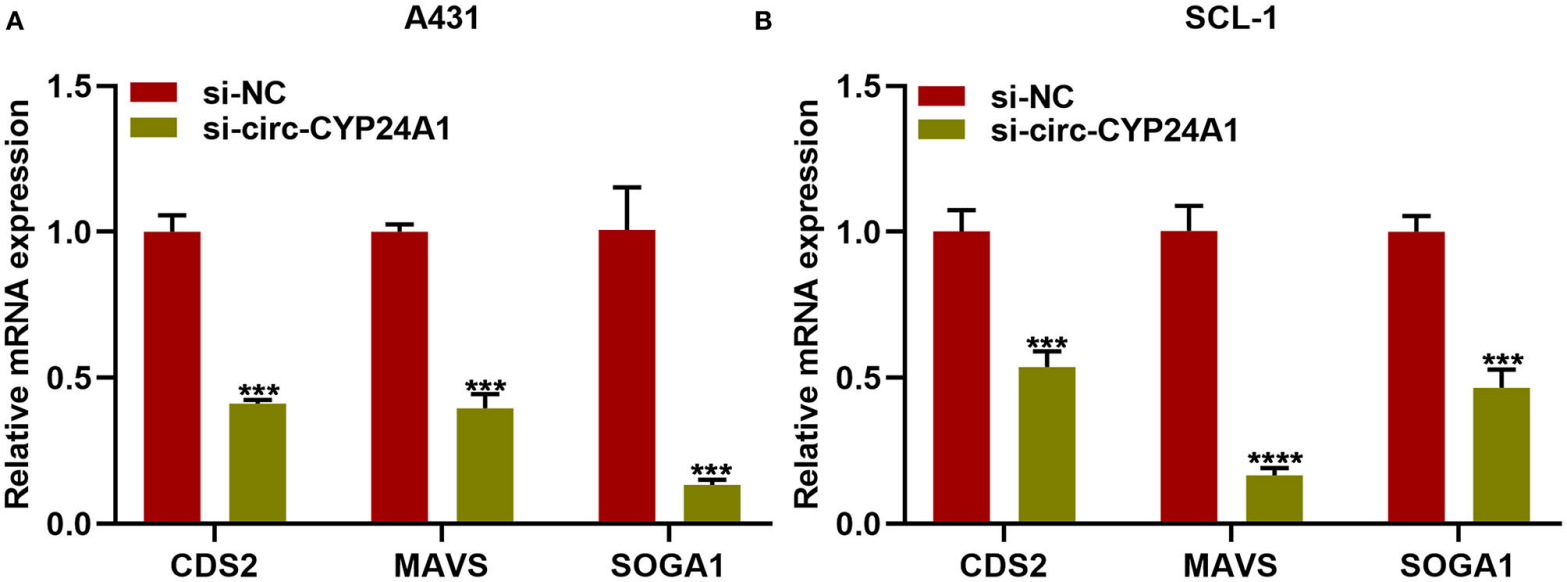
Exosomal circ-CYP24A1 knockdown lowers CDS2, MAVS and SOGA1 expression in cSCC cells. **(A,B)** RT-qPCR for the mRNA expression of CDS2, MAVS and SOGA1 in **(A)** A431 cells and **(B)** SCL-1 cells incubated with si-circ-CYP24A1-transfected exosomes. ***P-value < 0.001; ****p-value < 0.0001.

Our bioinformatics analysis predicted that CDS2, MAVS and SOGA1 were downstream mRNAs. Here, we further verified the relationships of exosomal circ-CYP24A1 with CDS2, MAVS and SOGA1. After incubation with si-circ-CYP24A1-transfected exosomes, RT-qPCR confirmed that the mRNA expression of CDS2, MAVS and SOGA1 was distinctly decreased in A431 cells (Figure 9A) and SCL-1 cells (Figure 9B). Above results indicated that CDS2, MAVS and SOGA1 could be potential targets of circ-CYP24A1 in cSCC.

## Discussion

This study constructed RNA-seq expression profiles of exosomal circRNAs in cSCC and verified the tumorigenic roles of exosomal circ-CYP24A1 in cSCC via regulating malignant biological behaviors.

CircRNA has a covalently closed loop structure without 5′ caps and 3′ poly tails. Several aberrantly expressed circRNAs have been reported. Circ-001937 is highly expressed in cSCC tissues (21). Its knockdown restrains proliferation as well as induces apoptosis through miRNA-597-3p/FOSL2 axis in cSCC (21). Circ-0070934 may facilitate proliferative and invasive capacities of cSCC cells through sponging miR-1238 and miR-1247-5p (22). Circ-0070934 promotes cSCC progression through miR-1236-3p/HOXB7 regulatory axis (23). Circ-0001821 up-regulation has been detected in cSCC tissues and induces proliferation as well as migration of cSCC cells (24). Circ-SEC24A is overexpressed in cSCC tissues and its silencing lowers proliferation, migration, invasion, and glycolysis as well as induces apoptosis in cSCC cells (25). Circ-0001360 is down-regulated in cSCC tissue specimens and up-regulating circ-0001360 may accelerate cSCC development (26). CircRNAs are enriched and stable in exosomes (27). There is distinct differentiation in exosomal circRNAs between patients and healthy individuals (28). Exosomal circRNAs have been considered as diagnosed markers for specific diseases (29). Nevertheless, the implications of exosomal circRNAs remain elusive in cSCC. Here, the differential expression of exosomal circRNAs was analyzed between 3 pairs of cSCC patients and healthy individuals by RNA-seq. 25 exosomal circRNAs were up-regulated and 76 were down-regulated in cSCC, which deserved in-depth exploration.

Exosomal circRNAs provide added evidence toward conventional diagnostic approaches, which are applied to restrain the malignant progression of malignant tumors (30). For instance, tumor-derived exosomal circ-PACRGL acts as the carcinogenic function in colorectal cancer progress and metastasis (31). Here, we verified a novel circ-CYP24A1 that was dysregulated in cSCC. Abnormal proliferation is a key factor in neoplastic transformation (32). Exosomal circ-CYP24A1 knockdown distinctly lowered the proliferative ability as well as elevated the apoptotic levels in cSCC cells. Despite the benign clinical behaviors, cSCC exhibits locally invasive and metastatic properties (33). Our data demonstrated that targeting exosomal circ-CYP24A1 may limit migrated and invasive capacities of cSCC cells. However, the clinical significance of exosomal circ-CYP24A1 will be validated in larger cSCC cohorts.

## Conclusion

Taken together, we systematically analyzed the exosomal circRNAs in cSCC and identified the carcinogenesis of exosomal circ-CYP24A1 in cSCC, which may provide a mechanistic insight into the roles of exosomal circRNAs in cSCC development and promising markers toward cSCC therapy.

## Data Availability Statement

The datasets presented in this study can be found in online repositories. The names of the repository/repositories and accession number(s) can be found below: https://www.ncbi.nlm.nih.gov/geo/query/acc.cgi?acc=GSE136113.

## Ethics Statement

The studies involving human participants were reviewed and approved by the study was approved by the Ethics Committee of The First Hospital of China Medical University (2018010). The patients/participants provided their written informed consent to participate in this study. Each participant signed the informed consent.

## Author Contributions

JL conceived and designed the study. ZZ conducted most of the experiments, data analysis, and wrote the manuscript. HG and WY participated in collecting data and helped to draft the manuscript. All authors reviewed and approved the manuscript.

## Conflict of Interest

The authors declare that the research was conducted in the absence of any commercial or financial relationships that could be construed as a potential conflict of interest.

## Supplementary Material

Supplementary Table 1 25 up-regulated circRNAs and 76 down-regulated circRNAs in cSCC compared to healthy subjects.

## Abbreviations

cSCC: cutaneous squamous cell carcinoma
circRNA: circular RNA
NTA: nanoparticle tracking analysis
TEM: transmission electron microscopy
FC: fold change
GO: Gene Ontology
KEGG: Kyoto Encyclopedia of Genes and Genomes
si-NC: si-negative control
CCK-8: Cell Counting Kit-8
TUNEL: Terminal deoxynucleotidyl transferase (TdT) dUTP Nick-End Labeling.
